# Agarolytic bacterium *Persicobacter* sp. CCB‐QB2 exhibited a diauxic growth involving galactose utilization pathway

**DOI:** 10.1002/mbo3.405

**Published:** 2016-12-17

**Authors:** Go Furusawa, Nyok‐Sean Lau, Appalasamy Suganthi, Abdullah Al‐Ashraf Amirul

**Affiliations:** ^1^ Centre for Chemical BiologyUniversiti Sains MalaysiaBayan LepasMalaysia; ^2^ Faculty of Earth ScienceUniversiti Malaysia KelantanJeliMalaysia; ^3^ School of Biological SciencesUniversiti Sains MalaysiaMindenMalaysia

**Keywords:** agarase, agarolytic bacterium, diauxic growth, leloir pathway, *Persicobacter*

## Abstract

The agarolytic bacterium *Persicobacter* sp. CCB‐QB2 was isolated from seaweed (genus *Ulva*) collected from a coastal area of Malaysia. Here, we report a high‐quality draft genome sequence for QB2. The Rapid Annotation using Subsystem Technology (RAST) annotation server identified four β‐agarases (PdAgaA, PdAgaB, PdAgaC, and PdAgaD) as well as galK, galE, and phosphoglucomutase, which are related to the Leloir pathway. Interestingly, QB2 exhibited a diauxic growth in the presence of two kinds of nutrients, such as tryptone and agar. In cells grown with agar, the profiles of agarase activity and growth rate were very similar. *galK, galE,* and phosphoglucomutase genes were highly expressed in the second growth phase of diauxic growth, indicating that QB2 cells use galactose hydrolyzed from agar by its agarases and exhibit nutrient prioritization. This is the first report describing diauxic growth for agarolytic bacteria. QB2 is a potential novel model organism for studying diauxic growth in environmental bacteria.

## 1 Introduction

Agar is a polysaccharide found in the cell walls of marine red algae, such as *Gelidium, Gracilaria, Gracilariopsis, Porphyra,* and *Pterocladia* (Renn, 1997). Agar is mainly composed of d‐galactose and an anhydro‐l‐galactose unit, which is alternately linked by β‐1,4‐ and α‐1,3‐glycosidic bonds (Araki, 1956). Agarases, agar‐degrading enzymes, are classified into two types, α‐agarase which cleaves α‐1,3‐linkages, and β‐agarase which cleaves β‐1,4‐linkages. The major products of these cleavages are agarooligosaccharides (Potin, Richard, Rochas, & Kloareg, 1993) and neoagarooligosaccharides (Aoki, Araki, & Kitamikado, 1990), respectively. These hydrolyzates exhibit several biological activities, including prebiotic, antioxidative, and immunopotentiating effects as well as skin‐moisturizing and whitening properties. In addition, a number of agarase have gained attention for important biotechnology applications. For example, Sugano and colleagues reported that agarase 0107 isolated from *Vibrio* sp. strain JT0107 digested agarose gel used for DNA electrophoresis, allowing 60% of the applied DNA to be recovered after incubating at 65°C for 5 min (Sugano, Terada, Arita, Noma, & Matsumoto, 1993).

Many agarolytic bacteria have been identified from seawater, marine algae, and marine sediments, including *Agarivorans* (Fu, Lin, & Kim, 2008; Lin et al., 2012), *Alteromonas* (Potin et al., 1993), *Catenovulum* (Cui, Dong, Shi, Zhao, & Zhang, 2014), *Cytophaga* (Duckworth & Turvey, 1969), *Flammeovirga* (Hou, Chen, Chan, & Zeng, 2015; Yang et al., 2011), *Microbulbifer* (Jonnadula & Ghadi, 2011; Miyazaki et al., 2008; Ohta et al., 2004), *Microscilla* (Naganuma, Coury, Polne‐Fuller, Gibor, & Horikoshi, 1993; Zhong et al., 2001), *Paenibacillus* (Mei et al., 2014), *Pseudoalteromonas* (Chi, Park, Kang, & Hong, 2014; Lu et al., 2009; Oh et al., 2010; Vera, Alvarez, Murano, Slebe, & Leon, 1998; Xavier Chiura & Kita‐Tsukamoto, 2000), *Pseudomonas* (Belas, Bartlett, & Silverman, 1988; Morrice, McLean, Williamson, & Long, 1983), *Pseudozobellia* (Nedashkovskaya et al., 2009), *Saccharophagus* (Ekborg et al., 2006), *Vibrio* (Sugano et al., 1993; Zhang & Sun, 2007), and *Zobellia* (Jam et al., 2005). These agarolytic bacteria metabolize agar primarily with β‐agarase. Several bacterial species, such as *Cytophaga flevensis* (van der Meulen, Harder, & Veldkamp, 1974) and *Saccharophagus degradans* (Ha et al., 2011), are able to use agar as a sole carbon source. To accomplish this exclusive agar metabolism, disaccharides and neoagarobioses produced by agarase are hydrolyzed to monomers, such as d‐galactose and 3,6‐anhydro‐l‐galactose. It has been reported that neoagarobiose hydrolase is essential for hydrolyzing neoagarobioses, which have been identified in *C. flevensis*,* Bacillus* sp., and *S. degradans* (Ha et al., 2011; Suzuki, Sawai, Suzuki, & Kawai, 2002; van der Meulen et al., 1974).

Three different galactose utilization pathways have been reported: Leloir pathway (Cardini & Leloir, 1953; Frey, 1996; Leloir, 1951), DeLey‐Doudoroff pathway (Ley & Doudoroff, 1957), and tagatose‐6‐P pathway (Nobelmann & Lengeler, 1996; Schneider, Jakel, Hoffmann, & Giffhorn, 1995). The Leloir pathway, commonly involved in galactose utilization, consists of galactokinase (GalK), galactose‐1‐phosphate uridylyltransferase (GalT), and UDP‐galactose‐4‐phosphate epimerase (GalE). The final product of the pathway, glucose‐1‐phosphate, enters the glycolytic pathway via a phosphoglucomutase reaction. Metabolism of d‐galactose by the DeLey‐Doudoroff pathway has been observed in *Escherichia coli* (Deacon & Cooper, 1977), *Gluconobacter liquefaciens* (Stouthamer, 1961), and *Azotobacter vinelandii* (Wong & Yao, 1994). This pathway generates d‐galactonate, 2‐keto‐3‐deoxy‐galactonate, and 2‐keto‐3‐deoxy‐6‐P‐galactonate as intermediary metabolites. The tagatose‐6‐P pathway found in *E. coli* and *Rhodobacter sphaeroides* involves the production and metabolism of galactitol and tagatose (Lai & Klapa, 2004). This pathway has been well studied in lactic acid bacteria. For example, *Lactococcus lactis* and *L. lactis* subsp*. cremoris* use both Leloir pathway and tagatose‐6‐P pathway. Thomas and colleagues reported that *L. lactis* metabolized galactose initially with the Leloir pathway, whereas *L. cremoris* initially used the tagatose‐6‐P pathway (Thomas, Turner, & Crow, 1980). Currently, galactose utilization pathways of agarolytic bacteria are poorly understood. One of our objectives in this study was to elucidate the galactose utilization pathway of agarolytic bacteria.

Genus *Persicobacter*, belonging to family *Flammeovirgaceae* is known to degrade agar, but has not yet been studied in detail. In this study, we describe *Persicobacter* sp. CCB‐QB2 (referred to here as QB2), a bacterium isolated from seaweed (genus *Ulva*), which exhibited diauxic growth in the presence of two nutrients, tryptone and agar. To date, at least, the diauxic growth of agarolytic bacteria has never been reported. Accordingly, to understand the diauxic growth of QB2, we first determined the whole‐genome sequence of QB2 and found four β‐agarases genes, Leloir pathway genes. Subsequently, quantitative reverse‐transcription PCR was conducted on the agarase and the Leloir pathway genes. Our results suggest that this bacterium metabolizes galactose with the Leloir pathway in the second growth phase of diauxic growth.

## 2 Experimental Procedures

### 2.1 Isolation and identification of *Persicobacter* sp. CCB‐QB2

*Persicobacter* QB2 was isolated from seaweed (genus *Ulva*) from Queens Bay of Penang Island, Malaysia, using a method described previously (Furusawa, Lau, Shu‐Chien, Jaya‐Ram, & Amirul, 2015). A piece of seaweed was transferred to a L‐ASWM (0.05% tryptone, 2.4% artificial sea water [ASW] with 10 mmol L^−1^ HEPES) agar plate. After incubating 2 days at 30°C, colonies exhibiting agarolytic activity formed a clear zone around the colonies. The colony were purified two times by single colony isolation on H‐ASWM (0.5% tryptone, 2.4% ASW with 10 mmol L^−1^ HEPES) agar plates (1.5% agar). QB2 cells grew to a density of 3 × 10^8^ cells ml^−1^ (OD_600_ = 1) in H‐ASWM at 30°C for overnight. Five microliters of the bacterial suspension was spotted on 1.5% agar H‐ASWM plates and incubated at 30°C for 24 hr. To confirm the agarolytic activity, this plate was stained with Lugol's solution (0.2 g iodine crystal and 2 g potassium iodine in 20 mL distilled water) (Cui et al., 2014).

Chromosomal DNA from mid‐log phase QB2 cells (3 × 10^8^ cells ml^−1^) was prepared using the Mygen Genomic DNA Prep Kit (Gene Xpress PLT). The protocol for phylogenetic analysis based on 16S rRNA gene sequence was described previously (Furusawa et al., 2015). The sequence data have been submitted to the DDBJ/EMBL/GenBank databases under the accession number KT285294.

### 2.2 Genome sequencing, assembly, and annotation

Chromosomal DNA from mid‐log phase QB2 cells (3 × 10^8^ cells ml^−1^) was prepared using the DNeasy Blood & Tissue Kit (QIAGEN). Whole‐genome sequencing for QB2 was performed with a PacBio RSII platform (Pacific Biosciences). A 10 kb Single Molecule Real‐Time (SMRT) bell library was prepared and sequenced using P5‐C3 chemistry according to the manufacturer's instructions. The library was sequenced using three SMRT cells, yielding 830 Mb of sequences from 117,232 reads, with an average read length of 7,081 bp. De novo assembly of the reads was performed following the Hierarchical Genome Assembly Process (HGAP) (v 2.2.0) workflow with default parameters (Chin et al., 2013). Using the workflow, the QB2 genome was assembled into two contigs of 3,843,562 and 31,064 bp, respectively. The genome was annotated using the Rapid Annotation using Subsystem Technology (RAST) server (Aziz et al., 2008) with default parameters. The genome sequence of the QB2 is available in DDBJ/EMBL/GenBank database under the accession number LBGV00000000.

### 2.3 Determination of growth curves and agarase activity

Two hundred microliters of a QB2 cell suspension (3 × 10^8^ cells ml^−1^) was transferred to 100 ml H‐ASWM, H‐ASWM with 0.2% agarose (Promega), L‐ASWM and L‐ASWM with 0.2% agarose. The samples were incubated at 30°C for 56 hr on a rotary shaker at 200 rpm. The growth of the bacterium at different incubation periods was measured by counting the colony‐forming units on H‐ASWM agar plates. Simultaneously, the agarase activity was also measured by the release of the reducing sugar equivalent using the 3,5‐dinitrosalicylic acid (DNS) method (Miller, 1959). One milliliter cell suspension was centrifuged and 10 μl of the supernatant was incubated in 90 μl of 20 mmol L^−1^ Tris‐HCl buffer (pH 7.6) containing 1.5% melted agarose at 50°C for 30 min. Subsequently, 200 μl DNS solution was mixed into the reaction solution and incubated at 100°C for 10 min. After heat treatment, 1 mL deionized water was added, and the absorbance of the reducing sugar was measured at 540 mm. The value was evaluated with d‐galactose as the standard. One unit (U) of enzymatic activity was defined as the amount of enzyme that released 1 μmol of reducing sugar per minute under this condition.

### 2.4 Quantitative real‐time PCR

Two hundred microliters of precultured QB2 cells (3 × 10^8^ cells mL^−1^) was transferred to 100 ml H‐ASWM, H‐ASWM with 0.2% agarose (Promega), L‐ASWM and L‐ASWM with 0.2% agarose. Samples were taken after 6, 9, and 30 hr incubation (roughly corresponding to the lag, first growth, and second growth phase observed for cells exhibiting diauxic growth). After harvesting cells, total RNA was extracted using TRIZOL Reagent (Ambion) and the QIAGEN RNeasy Mini Kit (QIAGEN) following the protocol described by Lopez and Bohuski (Lopez & Bohuski, 2007). cDNA was synthesized using the RevertAid H Minus First Strand cDNA Synthesis Kit (Thermo Scientific), and 20 μg of the resulting cDNA was added to a 20 μl PCR mixture prepared from Fast SYBR^®^ Green Master Mix (Applied Biosystems), which contained 10 μmol L^−1^ of each primer listed in Table S1 at a final concentration of 0.5 μmol L^−1^. The experiment was conducted in duplicate and three independent runs were performed for each experiment. The following thermal cycling parameters were used: denaturation at 95°C for 25 s, followed by 40 cycles of denaturation at 95°C for 3 s, annealing and extension at 60°C for 30 s. Melting curve analysis was conducted at temperature range of 45 to 95°C with a slope of 0.05°C per second. Fold change in gene expression was calculated for each gene in treated and control samples. All obtained data were normalized to the 16S rRNA gene.

## 3 Result and Discussion

### 3.1 Isolation, identification, and characterization of QB2

**Figure 1:**
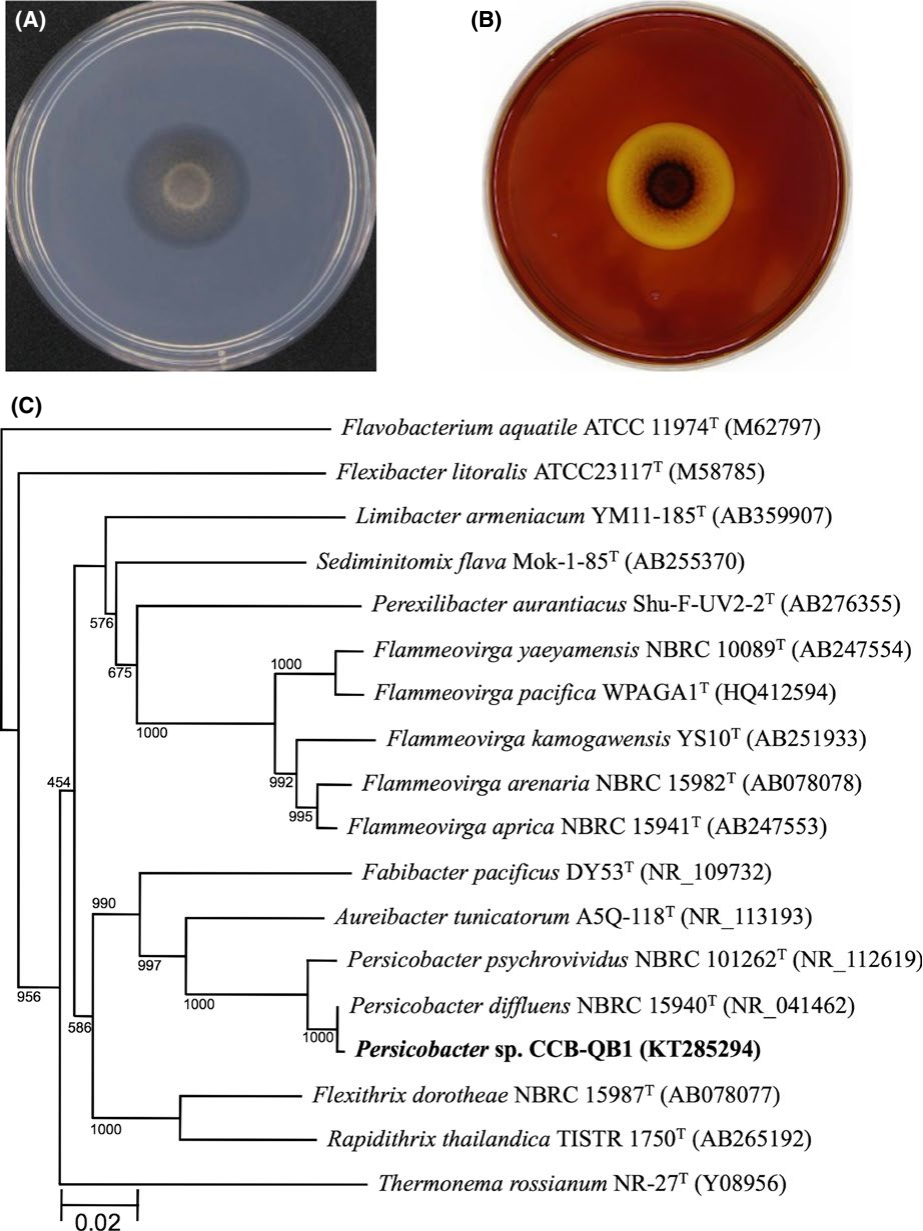
Characterization and phylogenetic analysis of QB2. (A) Colony morphology of QB2 grown on a H‐ASWM agar plate was observed using a Colony Doc‐It Imaging Station. (B) Agarolytic activity on the same plate was detected using Lugol's solution. (C) Phylogenetic tree based on 16S rRNA sequence from QB2 and related members of the *Bacteroidetes* phylum. The tree was constructed using the neighbor‐joining method (Saitou & Nei, 1987) of the ClustalW (v.2.1) program (Thompson, Higgins & Gibson, 1994). The numbers at the nodes indicate bootstrap confidence values obtained after 1000 replications. The scale bar indicates genetic distance

An agar‐degrading bacterium was isolated from seaweed (genus *Ulva*) obtained from a coastal region of Penang, Malaysia. When grown on agar plate, this bacterium formed a salmon‐pink pigmented colony and exhibited swarming behavior by gliding motility (Fig. 1A). A clear zone formed by agar degradation was clearly visualized by staining with Lugol's solution (Fig. 1B). The 16S rRNA gene sequence from the isolate was identical to *Persicobacter diffluens* strain NBRC 15940. A phylogenetic tree was prepared from 16S rRNA sequences from several members of the *Bacteroidetes* (Fig. 1C), and this isolate clustered with *P. diffluens*. Genus *Persicobacter* in the family *Flammeovirgaceae* consists of two species, *P. diffluens a*nd *P. psychrovividus* (Muramatsu et al., 2010), both of which are known to degrade agar. Based on the apparent relatedness to this genus, the isolate was tentatively named, *Persicobacter* sp. CCB‐QB2 (Centre for Chemical Biology‐Queens Bay 2).

### 3.2 QB2 cells exhibited a diauxic growth

To estimate the agarase production of QB2, the cells were cultured in two media, high‐nutrient artificial seawater medium (H‐ASWM) and low‐nutrient artificial seawater medium (L‐ASWM) with or without 0.2% agarose.

**Figure 2:**
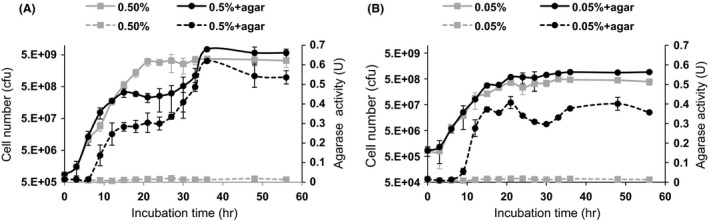
Cell growth (colony‐forming units) and agarase activity of QB2. QB2 cells cultured in H‐ASWM (0.5% tryptone) with or without 0.2% agarose (A) or L‐ASWM (0.05% tryptone) with or without 0.2% agarose (B). Gray and black solid lines indicate cell numbers in the sample without agar and the sample with agar, respectively. Gray and black dash lines indicate agarase activities in the sample without agar and the sample with agar, respectively. All data shown are mean values from three replicate experiments. Error bars denote the standard deviation of triplicate samples

As shown in Fig. 2A, QB2 cells grown in H‐ASWM (0.5% tryptone) with and without agar exhibited exponential growth after 3 hr. Cell growth in H‐ASWM without agar reached stationary phase at 21 hr. In contrast, although growth of cells in H‐ASWM with agar exhibited a stationary phase after 15 hr, these cells exhibited a second growth phase after 30 hr. In the first stationary phase, the cell number in H‐ASWM with agar was approximately 11 times lower than that observed in H‐ASWM without agar. However, the final cell number in H‐ASWM with agar was two times higher (7.5 × 10^9^ cells ml^−1^) than that of cells grown in H‐ASWM without agar (Fig. 2A). Thus, QB2 exhibits diauxic growth in the presence of both tryptone and agar. This ability was dependent on the concentration of tryptone: in media containing 0.05% tryptone (L‐ASWM) and agar, QB2 cells did not exhibit diauxic growth; however, the cell number in L‐ASWM with agar was approximately two times higher than that of L‐ASWM without agar (Fig. 2B). These results suggest that QB2 cells metabolize agar as nutrient with tryptone and exhibit better growth than that of without agar. The use of media supplemented with agar has previously reported in studies to optimize the agarase production (Hu, Lin, Xu, Zhong, & Liu, 2009; Leon, Quintana, Peruzzo, & Slebe, 1992; Potin et al., 1993; Sugano et al., 1995; Vera et al., 1998; Xavier Chiura & Kita‐Tsukamoto, 2000). However, diauxic growth was not reported in any of these cases.

Agarase activity was detected after 9 hr incubation in both high‐ and low‐nutrient conditions with agar (Fig. 2A and B). In the first stationary phase, the agarase activity of L‐ASWM with agar was slightly higher (about 1.3 times) than that of H‐ASWM with agar. However, in second stationary phase, agarase activity in H‐ASWM with agar was twofold higher than that L‐ASWM with agar, reaching a maximum of 0.62 unit ml^−1^ (Fig. 2A). The curves for agarase activity and growth rates were similar for cells grown in agar. The agarase activity of cells grown without agar remained in lower level (0.014 unit ml^−1^) throughout incubation (Fig. 2A and B).

### 3.3 General genome features

To understand the agarolytic process, galactose utilization pathway and the diauxic growth, whole‐genome shotgun sequencing of QB2 was carried out using PacBio single molecule real‐time (SMRT) sequencing technology, a third‐generation sequencing technology that does not require an amplification step prior to sequencing. The hierarchical genome‐assembly process (HGAP) which has been shown to produce near‐finished high‐quality assemblies from only long reads produced by the PacBio system (Chin et al., 2013), was used. The final genome assembly of QB2 consisted of two contigs with a total GC content of 42.5% (Fig. S1). The GC content is quite similar to that of the type strain *P. diffluens* (42.6–43.8%) (Muramatsu et al., 2010). Approximately, 99% of the genome assembly is contained within one large 3.84‐Mb contig. The genome contains 3,693 protein‐encoding genes and 166 RNA genes. Of the total 3,693 protein‐encoding genes, 1889 were assigned to a putative function, and the remaining were annotated as hypothetical proteins (Table 1).

**Table 1:** Properties of the high‐quality draft genome sequences of QB2

Attribute	Value
Genome size (bp)	3.84 Mbp
DNA G+C content (%)	42.5%
Number of contigs	2
Total protein‐encoding genes	3,693
Total RNA genes	51
Genes assigned a putative function	1,889
Genes annotated as hypothetical proteins	1,804

### 3.4 QB2 agarases

Based on amino acid sequence similarities, putative genes assigned β‐agarases function have been classified into four families: GH16, GH50, GH86, and GH118 (Cui et al., 2014; Martin, Portetelle, Michel, & Vandenbol, 2014). Four agarases (PdAgaA, PdAgaB, PdAgaC, and PdAgaD) were identified in the QB2 genome using the Rapid Annotation using Subsystem Technology (RAST) annotation server while two agarases were found from *Persicobacter* sp. JZB09 (Han et al., 2012). Although the amino acid sequences of PdAgaA, PdAgaB, and PdAgaC exhibited a high degree of similarity to AgaG4 from *F. yaeyamensis* MY04 (47.9%), AgaA from *Pseudomonas* sp. CY24 (40.2%), and AgaP4383 from *F. pacifica* WPAGA1 (67.3%), the similarity of PdAgaD to other known agarases was relatively low (less than 40%) (Table 2).

**Table 2:** Agarase genes of QB2 predicted by RAST annotation

Gene product	Location	Module	Function predicted by RAST	Similarity
PdAgaA	895296_892912	GH86, Ricin B	β‐agarases precursor	47.9% compared to AgaG4 from *F. yaeyamensis* MY04
PdAgaB	897631_895787	GH16, Ricin B, PorSSSM	Agarase	40.2% compared to AgaA from *Pseudomonas* sp. CY24
PdAgaC	1219317_1217380	GH16, CBM6, PorSSSM	Agarase	67.3% compared to AgaP4383 from *F. pacifica* WPAGA1
PdAgaD	1728593_1726944	GH16, Ricin B, PorSSSM	Agarase	Less than 40% compared to other known agarases

**Figure 3:**
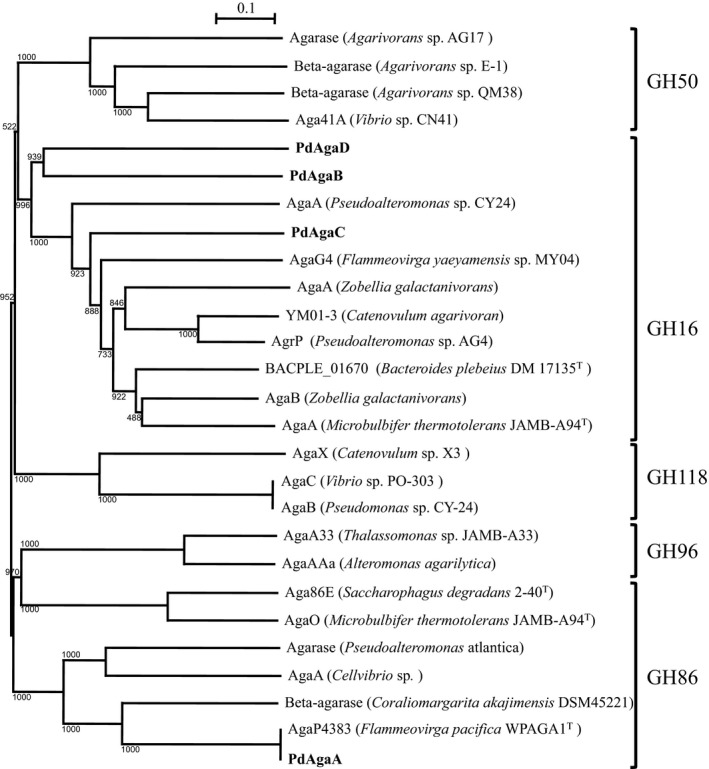
Phylogenetic analysis of QB2 agarases PdAgaA, PdAgaB, PdAgaC, and PdAgaD with known β‐agarases classified by the CAZy database. The tree was constructed with the neighbor‐joining method from the ClustalW (v.2.1) program. The numbers indicate the bootstrap confidence values obtained after 1,000 replications. The scale bar indicates the genetic distance

Manual BLASTP search and phylogenetic analysis suggested that PdAgarA belongs to the GH86 family of agarase and PdAgaB, PdAgaC, and PdAgaD belong to the GH16 family (Fig. 3), which is widely distributed in marine bacteria (Cui et al., 2014). Structural analysis reported by Allouch and colleagues indicated that the GH16 modules of AgaA and AgaB from *Z. galactanivorans* contain two essential catalytic site residues, the nucleophilic residue Glu147 and acid/base residue Glu152, in addition to the Asp‐149 that may play an important role in maintaining charges in the environment of the catalytic amino acids (Allouch et al., 2003). Similarly, the GH16 modules in PdAgaB, PdAgaC, and PdAgaD of QB2 also contained a conservative catalytic motif, ExDxxE (Fig. S2). Residues binding calcium ions were also found in AgaA and AgaB sequences, and these residues were conserved in PdAgaB, PdAgaC, and PdAgaD (Fig. S2).

The GH86 family contains of five agarases isolated from *P. atlantica* T6c (Morrice et al., 1983), *Cellvibrio* sp. (AgaA)(Ariga et al., 2012), *M. thermotolerans* JAMB‐A94 (AgaO) (Ohta et al., 2004), *S. degradans* 2‐40 (Aga86E) (Ekborg et al., 2006), and *F. pacifica* WPAGA1 (AgaP4383) (Hou et al., 2015). Three agarases, AgaO, Aga86E, and AgaP4383, possessed a carbohydrate‐binding module (CBM) from the CBM6 family, including calcium‐binding sites. However, the CBM6 family module was not found in PdAgaA.

PdAgaA and PdAgaB sequences also contain the ricinB_lectin_2 domain (Pfam:PF14200), which has been identified as a noncatalytic domain from fungus that binds Gal‐α‐(1,3)‐Gal‐ β‐(1,4)‐GlcNAc (Grahn et al., 2007), Neu5Ac‐ α‐(2‐6)‐Gal (Kadirvelraj et al., 2011), Gal‐ β‐(1,3)‐GalNAc (Sulzenbacher et al., 2010), and Gal‐ β‐(1,3)‐GalNAc (Pohleven et al., 2012). The domain contains three highly conserved (Q‐x‐W)_3_ motifs that are found in the three subdomains and are comprised of a motif of four β‐strands repeated three times, respectively. The ricinB_lectin_2 domain of both agarases contains (Q‐x‐W)_3_ motifs in three subdomains (Fig. S3). BLASTP search results suggested that PdAgaD also has a ricinB_lectin_2 domain, although (Q‐x‐W)_3_ motifs were not found. Cui and colleagues reported that β‐agarase YM01‐3 isolated from *C. agarivorans* YM01T includes the ricinB_lectin_2 domain (Cui et al., 2014). However, the function of this domain in the agarase is not yet understood.

PdAgaC, belonging to the GH16 family, contains a CBM6 family module. The noncatalytic carbohydrate modules linked to the catalytic modules have been shown to be associated with plant cell wall‐degradation enzymes, such as xylanase (Boraston, Bolam, Gilbert, & Davies, 2004; Czjzek et al., 2001) and glucanase (Pires et al., 2004). CBMs play a critical role in enhancement of catalytic efficiency to bind the insoluble forms of the polysaccharides (Gill et al., 1999). A β‐sandwich fold in the CBM6 comprised two β‐sheets, each consisting of three to six antiparallel β‐strands (Boraston et al., 2004). For example, the β‐sandwich fold of Aga16B‐CBM6‐2 from *S. degradans* contains two β‐sheets: one with five β‐strands and one with six β‐strands (Henshaw et al., 2006). Secondary structure of CBM6 from PdAgaC as predicted by the self‐optimized prediction method (SOPM) (Geourjon & Deléage, 1994) contained eight β‐strands (Fig. S4). Henshaw and colleagues have reported the crystal structures and functions of several CBM6s identified in GH16 family agarases from *S. degradans* 2‐40. The substrate‐binding sites that are found in Aga16B‐CBM6 of *S. degradans* 2‐40 are partially conserved in CBM6 of PdAgaC (Fig. S4) (Henshaw et al., 2006).

In addition, PdAgaB, PdAgaC, and PdAgaD contained Por secretion system (PorSS) sorting modules. The PorSS is a novel protein secretion system identified in *Flavobacterium johnsoniae* and *Porphyromonas gingivalis* in the *Bacteroidetes* phylum. It was reported that the PorSS of *F. johnsoniae* is related to assembly of the gliding motility apparatus and secretion of chitinase (Rhodes et al., 2010) and that PorSS of *P. gingivalis* is needed for secretion of gingipain proteases, which are major virulence factors of periodontal pathogenesis (Sato et al., 2010). The PorSS consists of seven genes, *gldK*,* gldL*,* gldM*,* gldN*,* sprA*,* sprE,* and *sprT*, and these genes are widely distributed in the *Bacteroidetes* (McBride & Zhu, 2013). BLASTP search indicated that these genes are also present in the QB2 (Table 3).

**Table 3:** Genes of Por secretion system predicted from the genome of QB2

Gene	Location	MW (kDa)	Predicted function by RAST
*gldK*	3568245_3569246	38.0	GldJ
*gldL*	3569296_3570171	31.1	Hypothetical protein
*gldM*	3570201_3571814	58.3	Gliding motility‐related protein
*gldN*	3571833_3572663	31.9	Hypothetical protein
*sprA*	3076837_3083976	270.7	Hypothetical protein
*sprE*	3579663_3577000	102.2	TPR domain protein
*sprT*	232573_233289	26.8	PorT protein

It has been reported that ZgAgaA from *Z. galactanivorans* (Jam et al., 2005) and AgaG4 from *F. yaeyamensis* (Han et al., 2013) also contain PorSS sorting modules (PorSSSM). However, these two agarases also contain type I signal peptides at the N‐terminus of the proteins. Jam and colleagues reported that the signal peptide and C‐terminal region containing PorSSSM were not present in the matured ZgAgaA protein (Jam et al., 2005). These observations suggest that secretion of ZgAgaA was regulated by two maturation steps, involving removal of the signal peptide from the N‐terminal end and cleavage of the PorSSSM from C‐terminal region. However, these signal peptides are not present in PdAgaB, PdAgaC, and PdAgaD, suggesting that these proteins might be secreted only by PorSS.

In agar metabolism, hydrolysis step of neoagarobioses is critically important for releasing d‐galactose and 3,6‐anhydro‐l‐galactose. Rebuffer and coauthors reported that 1,3‐α‐3,6‐anhydro‐l‐galactosidase discovered from *Zobellia galactanivorans* cleaves α‐1,3 glycosidic bond of neoagarobioses (Rebuffet et al., 2011). This protein belongs to GH117 family, which is distantly related to GH43 family. In this study, although GH117 family proteins were not identified from the QB2 genome, a GH43 family protein was found in the QB2 genome. GH43 and GH117 contain five‐bladed β‐propeller fold and positions of catalytic residues are similar between GH43 and GH117. Hence, it is likely that a GH43 family protein present in QB2 genome might be substituting for GH117 family protein.

### 3.5 Galactose utilization pathway of QB2

The fact that QB2 cells exhibit diauxic growth in the presence of agar and tryptone suggests that QB2 cells might be metabolizing d‐galactose resulting from the hydrolysis of agarose. To identify the galactose‐metabolizing pathway used in QB2, a sequences similarity search was performed with the QB2 genome using gene sequence from Leloir, DeLey‐Doudoroff, and tagatose‐6‐P galactose utilization pathways (Lai & Klapa, 2004). Although genes related to the Leloir pathway were identified from QB2 genome (Table 4), genes related to DeLey‐Doudoroff and tagatose‐6‐P galactose utilization pathways were not detected.

**Table 4:** Leloir pathway gene sequences identified in the genome of QB2

Gene product	Location	Function predicted by RAST	Similarity to Flammeovirga genes (%)
GalK	3589447_3588302	Galactokinase	74.8
GalE	268264_269190	UDP‐galactose‐4‐epimerase	71.1
Phosphoglucomutase	2153993_2152263	Phosphoglucomutase	76.3

The pathway consists of galactokinase (GalK), galactose‐1‐phosphate uridylyltransferase (GalT), UDP‐4‐galactose epimerase (GalE), and phosphoglucomutase. In the Leloir pathway, which has been well studied for enterobacteria, especially lactic bacteria (Grossiord, Vaughan, Luesink, & de Vos, 1998; Thomas et al., 1980), galactose is initially phosphorylated by GalK to galactose‐1‐phosphate. Subsequently, galactose‐1‐phosphate is converted to glucose‐1‐phosphate via UDP‐glucose by GalT. UDP‐glucose is produced from UDP‐galactose through the GalE. Finally, phosphoglucomutase catalyzes the conversion of glucose‐1‐phosphate to glucose‐6‐phosphate, which corresponds to the initial step of the glycolysis and pentose phosphate pathways. GalK, GalE, and phosphoglucomutase sequences identified from the QB2 genome exhibited 74.8% similarity to *Flammeovirga pacifica* GalK, 71.1% similarity to *Flammeovirgaceae bacterium 311* GalE, and 76.3% similarity to *Flammeovirgaceae bacterium 311* phosphoglucomutase, respectively.

No sequences significantly similar to GalT were identified from the QB2 genome, however, a HIT (histidine triad) protein which exhibits very low coverage and similarity to *F. pacifica* GalT was found in the QB2 genome. HIT protein can be divided into three branches, HINT (histidine triad nucleotide‐binding protein), FINT (fragile histidine triad protein), and GalT. Although the sequence similarity between HINT and GalT are quite low, the structure of GalT monomer and HINT dimer can be superimposed. Moreover, some of the same residues consisting of their active sites are retained (Brenner, 2002). It is likely that a HIT protein present in QB2 genome might be substituting for GalT.

### 3.6 Expression of agarase genes according to growth phase

**Figure 4:**
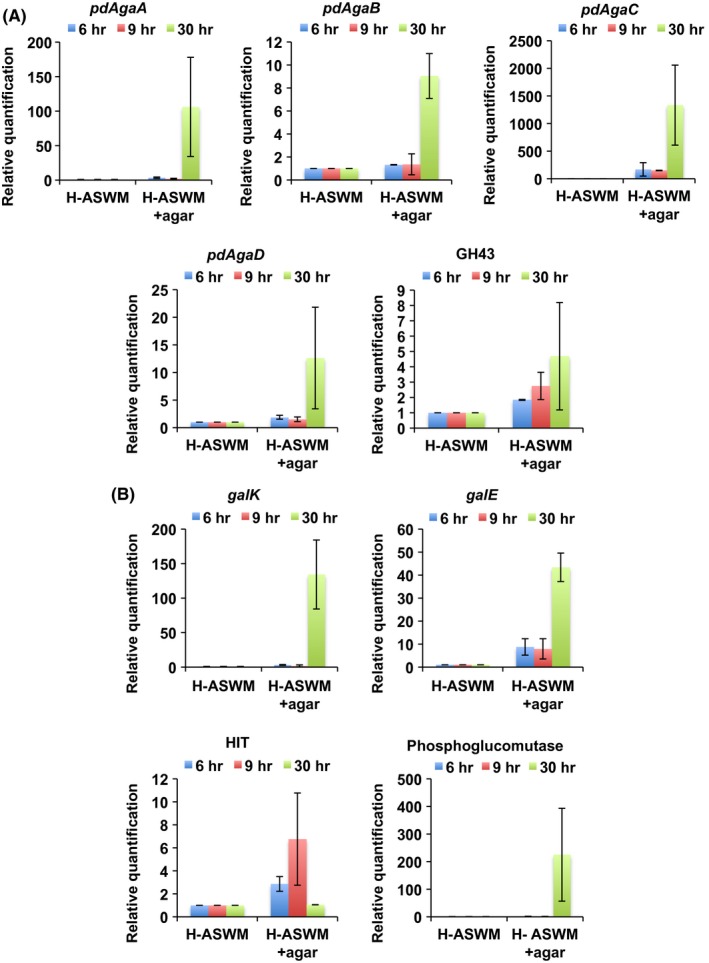
Relative expression levels of four agarases, *pdAgaA*,* pdAgaB*,* pdAgaC,* and *pdAgaD* (A) and *galK* and phosphoglucomutase gene (B) during lag (6 hr), first growth (9 hr), and second growth (30 hr) phases from cells cultured in same medium in Fig. 2. The 16S rRNA gene was used as an endogenous control. Data are the mean of two replicate experiments. Errors indicate the standard deviation of duplicate samples

Quantitative reverse‐transcription PCR (qRT‐PCR) was performed to evaluate the relative expression of agarase genes during each phase (lag, first growth, and second growth) observed for cells exhibiting diauxic growth. As shown in Fig. 4A, *pdAgaC* exhibited a high level of gene expression than that of the other three agarases and was particularly highly expressed in cells grown in H‐ASWM with agar: in the second growth phase, the expression level of *pdAgaC* was approximately 1300 times higher than that of cells in H‐ASWM without agar. The highest levels of *pdAgaA* expression were observed in H‐ASWM with agar in the second growth phase and 100‐fold of that of the H‐ASWM without agar. *pdAgaB* and *pdAgaD* expression was detected mainly in the second growth phase in H‐ASWM with agar samples. Taken together, these results indicate that PdAgaC might play primary role in agarase activity in QB2 under these conditions.

As mentioned above, it is speculated that the GH43 family protein found in QB2 genome functions instead of GH117 family protein in the final step of agar metabolism. To confirm the hypothesis, qRT‐PCR was also performed to confirm the gene expression of the GH43 family protein. The highest levels of gene expression of the GH43 family protein were observed in H‐ASWM with agar in the second growth phase as well as *pdAgaA*,* pdAgaB*,* pdAgaC,* and *pdAgaD* and 4.7‐fold of that of the H‐ASWM without agar (Fig. 4A), suggesting that the GH43 family protein might has alternative function to GH117 family protein.

### 3.7 Expression of genes for Leloir pathway during growth

Based on high agarolytic activity and high level of expression of agarase genes at the second growth phase in QB2 cells as well the presence of gene sequences related to the Leloir pathway in the genome, we hypothesized that QB2 metabolizes agar during the second growth phase. To test this hypothesis, expression of *galK, galE,* and phosphoglucomutase genes was examined using qRT‐PCR. As shown in Fig. 4B, the expression levels of *galK, galE,* and phosphoglucomutase was low during the lag phase (6 hr) and first growth (9 hr) for cells grown in H‐ASWM with and without agar. However, at second growth phase (30 hr), the expression levels of *galK, galE,* and phosphoglucomutase gene were 134, 43, and 225 times higher in cells grown in H‐ASWM with agar than for cells in H‐ASWM without agar, respectively. These results suggested that tryptone metabolism may be prioritized over agar at first growth phase and that galactose hydrolyzed from agar might be used during the second growth phase in the presence of agar.

In addition, gene expression of HIT protein which might be substituting for GalT was also tested. During the lag phase (6 hr) and first growth (9 hr), the expression level of HIT gene was 2.9 and 6.8 times higher in cells grown in H‐ASWM with agar than for cells in H‐ASWM without agar. However, at second growth phase (30 hr), the expression level of HIT gene was quite similar to that for cells in H‐ASWM without agar (Fig. 4B).

Results of qRT‐PCR showed that agarase genes and genes for Leloir pathway without HIT gene were highly expressed at second growth phase in H‐ASWM with agar, but HIT gene was not highly expressed at second growth phase in H‐ASWM with agar in comparison with the gene expression in cells grown in H‐ASWM without agar. As a possibility, low level of HIT gene expression at second growth phase may be a limiting factor in cell growth. Accordingly, although QB2 cells showed diauxic growth in the presence of agar, the cell number of the sample with agar in stationary phase was only two times higher than that of the sample without agar.

In conclusion, QB2 cells exhibited diauxic growth and nutrient prioritization. Agarase genes and genes for Leloir pathway without HIT gene were highly expressed in second growth phase of the diauxic growth, suggesting galactose metabolism might support the cell growth in second growth phase. Thus, QB2 is a potential novel model organism for studying diauxic growth of in environmental bacteria.

## Funding Information

We are grateful to Universiti Sains Malaysia for funding this project (1001/PCCB/870009) and also for postdoctoral position fellowships granted to Go Furusawa and Nyok‐Sean Lau.

## Conflict of Interest

None declared.
