# Supplementary material for: Agarolytic bacterium Persicobacter sp. CCB‐QB2 exhibited a diauxic growth involving galactose utilization pathway
Source: Microbiologyopen. 2016 Dec 17;6(1):e00405. doi: 10.1002/mbo3.405 (PMC5300873; doi:10.1002/mbo3.405)
Supplement: Supplementary file 1 [file MBO3-6-0-s001.pdf]

Supporting Information

Table S1: Primer sequences used for qPCR.

Primer name	Primer Sequences (5'-3')
PdAgaA	F- CGGAGCGATGACTCTGGAAA<br>R- GTTGCGTCCAAGGGTAGGAA
PdAgaB	F- ATTAAAGGCCACCTGGGACG<br>R- CCATTTTTGGTGGGAACCGC
PdAgaC	F- CGGATCCGGGAAATGGGAAA<br>R- CCATCCCAGCTGTTATGCCA
PdAgaD	F- CCAATGCCAGTGCTTTCCAC<br>R- AGGAGCGGTAAAAGGAACCG
Galactokinase	F- GCGTGATGTTTCCCTTGA<br>R- GCAGCTTCTACTCGGTTATT
Phosphoglucomutase	F- CTTCCCGACTGTGGTTTATC<br>R- GTGATTCTTCACCGCTATCC
16s rRNA	F- TGAGCGACGGTTCCAGAAAT<br>R- AGTTCTGTTAGAGTCCCCAGCATT

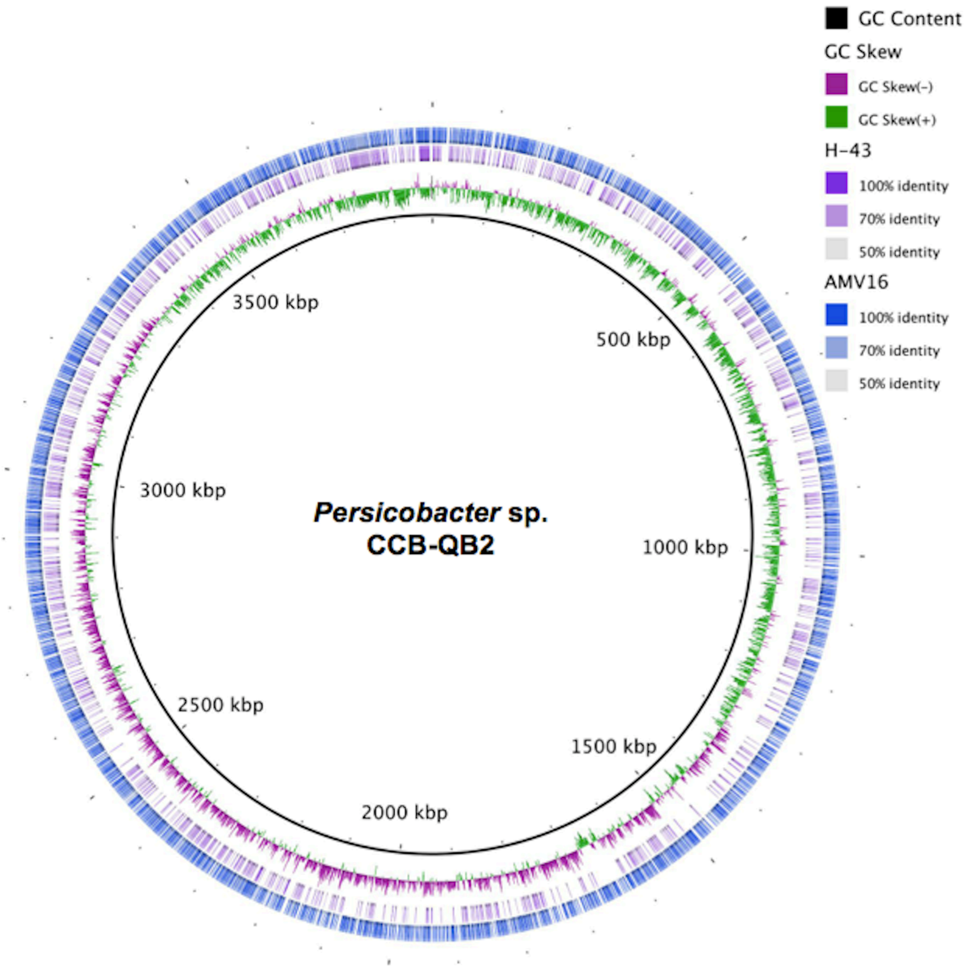

Fig. S1 Genome-wide comparison of the QB2, *Marivirga tractuosa* H-43^{T} and *Cesiribacter andamanensis* AMV16^{T} genomes was performed using the BLAST RING Image Generator (BRIG) (1). The *C. andamanensis* genome assembly was ordered against the QB2 genome assembly using Mauve (2). From outside to the center: BLAST comparison of the *C. andamanensis* and QB2 (blue), BLAST comparison of the *M. tractuosa* genome and QB2 genome (purple), GC skew (purple and green) and GC content (black).

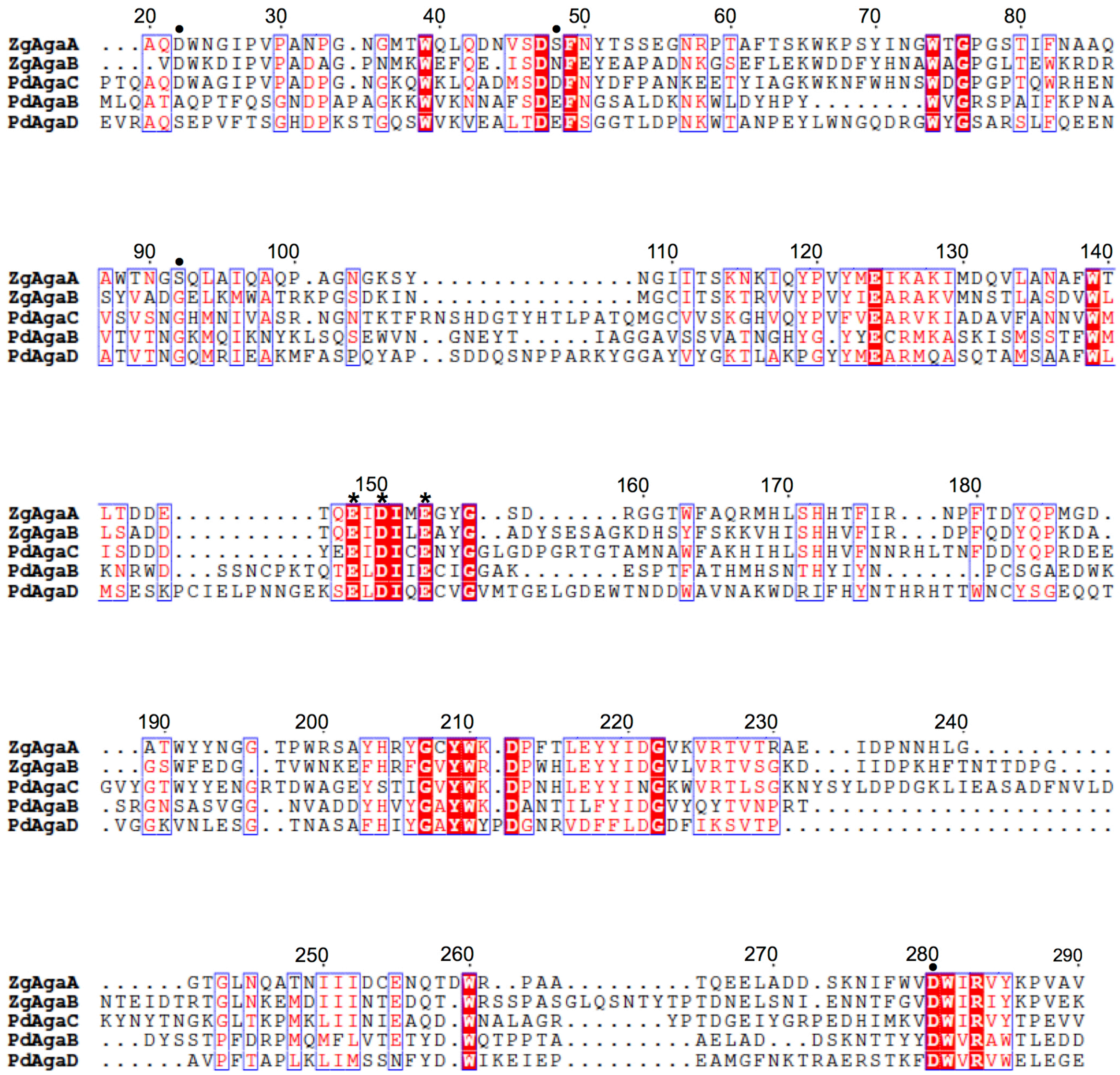

Fig. S2. Alignment of amino acid sequences from GH16 domains PdAgaB, PdAgaC and PdAgaD and *Z. galactanivorans* sequences AgaA and AgaB (labeled as ZgAgaA and ZgAgaB, respectively). In the *Z. galactanivorans* sequences, asterisks indicate the catalytic residues and black circles indicate the residues involved in calcium binding. The figure was drawn using the program ESPript program (3).

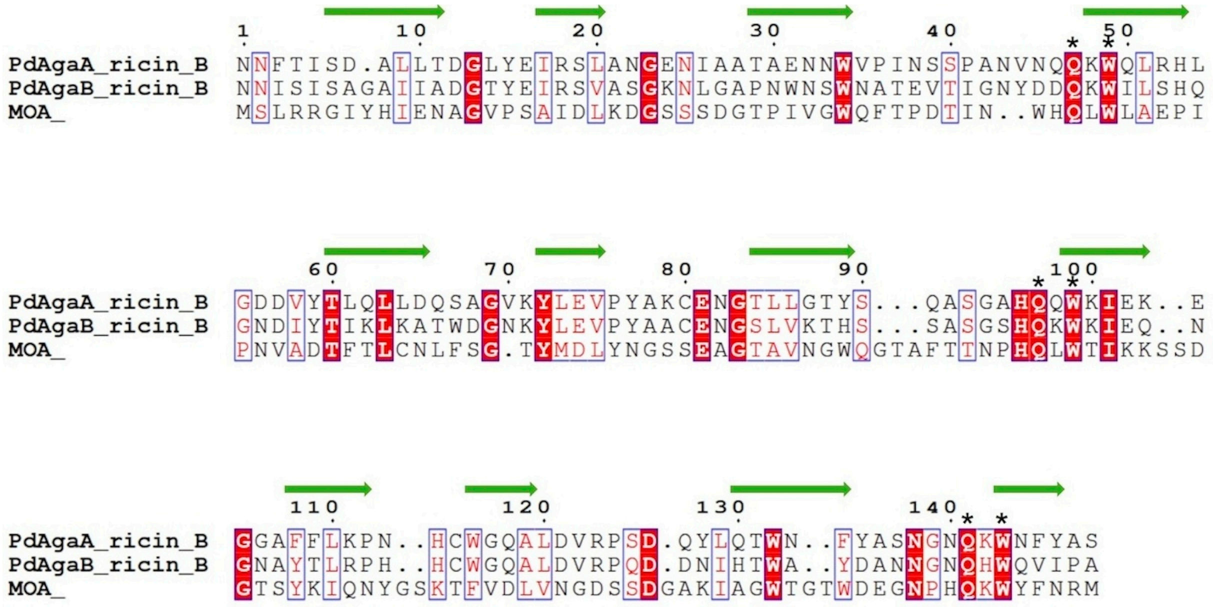

Fig. S3. Alignment of amino acid sequences of ricinB_lection_2 domains from PdAgaA and PdAgaB and from *Marasmius oreades* agglutinin (labeled as MOA). Asterisks indicate highly conserved (Q-x-W)$_3$ motifs found in MOA. Green arrows indicate β–strands. The figure was drawn using the program ESPript program.

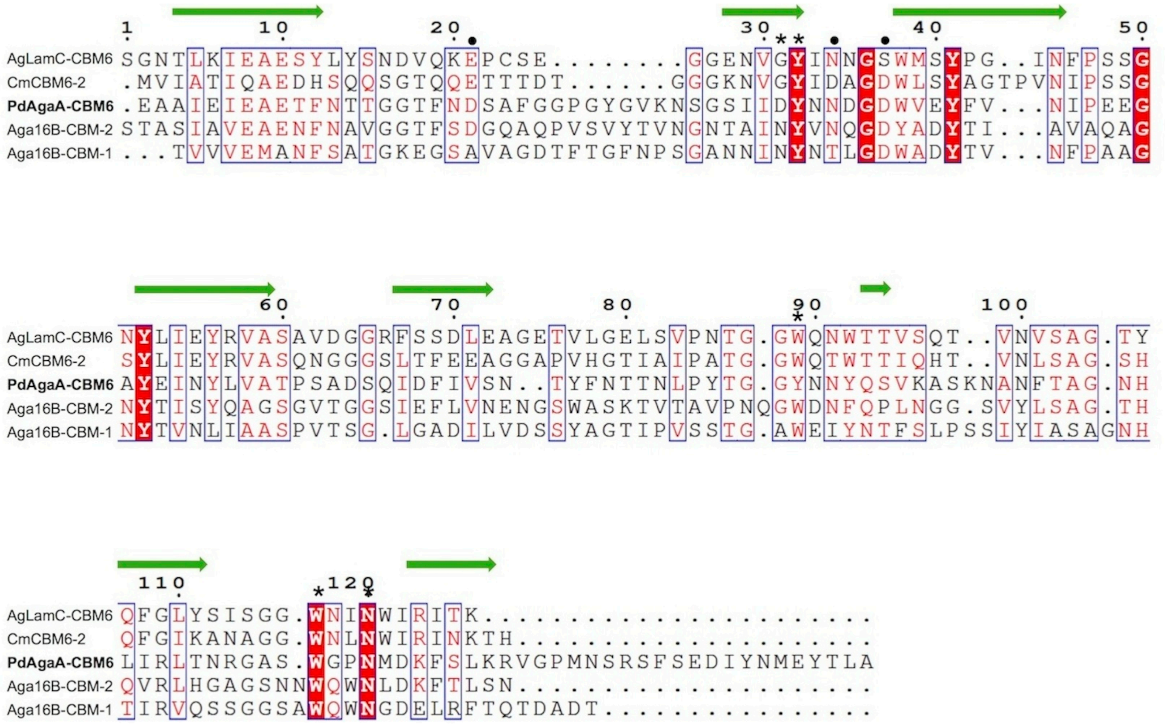

Fig. S4. Alignment of amino acid sequences from CBM6 domains from PdAgaC with *Z. galactanivorans* laminarinase (labeled as AgLamC-CBM6), *C. mixtus* lichenase 5a (labeled as CmCBM6-2) and *S. degradans* Aga16B (labeled as Aga16B-CBM-1 and Aga16B-CBM-2). For the *S. degradans* sequences, asterisks indicate the catalytic residues and black circles indicate the residues involved in calcium binding. Green arrows indicate β–strands. The figure was drawn using the program ESPript program.

Supplemental references

1. Alikhan N-F, Petty NK, Zakour NLB, Beatson SA. BLAST Ring Image Generator (BRIG): simple prokaryote genome comparisons. BMC Genomics. 2011;12(1):402 DOI: 10.1186/471-2164-12-402.

2. Darling ACE, Mau B, Blattner FR, Perna NT. Mauve: multiple alignment of conserved genomic sequence with rearrangements. Genome Research. 2004;14(7):1394-403.

3. Robert X, Gouet P. Deciphering key features in protein structures with the new ENDscript server. Nucleic Acids Research. 2014;42(W1):W320-W4.
